# The prevalence of metabolic syndrome and cardiovascular risk factors in a group of obese Saudi children and adolescents: A hospital-based study

**DOI:** 10.4103/0256-4947.55164

**Published:** 2009

**Authors:** Doris Taha, Omaima Ahmed, Bakr bin Sadiq

**Affiliations:** a From the Department of Pediatrics, King Faisal Specialist Hospital and Research Centre, PO Box 40047, Jeddah 21499, Saudi Arabia; b From the Research Centre, King Faisal Specialist Hospital and Research Centre, PO Box 40047, Jeddah 21499, Saudi Arabia

## Abstract

**BACKGROUND AND OBJECTIVES:**

We assessed the distribution of risk factors associated with the metabolic syndrome in a group of obese Saudi children and adolescents. No previous studies had addressed this issue in the Saudi pediatric population.

**SUBJECTS AND METHODS:**

We retrospectively reviewed the medical records of patients evaluated for obesity between 2004 and 2008 and collected data on age, weight, height, body mass index (BMI), BP, fasting lipid profile, fasting glucose, insulin concentrations, and insulin resistance based on the homeostasis assessment model-insulin resistance (HOMA-IR) score. Obesity was defined as a BMI above the 95th percentile for age and gender and metabolic syndrome was diagnosed according to standard criteria.

**RESULTS:**

We studied 57 obese Saudi children and adolescents with a mean (standard deviation) age of 9.8 (3.5) years. Mean weight and body mass index (BMI) were 63.7 (28.3) kg and 31.6 (8.0) kg/m^2^, respectively. Systolic BP was elevated in 24 (42%) of the 57 subjects. Of the 39 children who had a lipid profile in their records, 10 had hypertriglyceridemia, 8 had hypercholesterolemia, 6 had elevated LDL cholesterol levels, and 6 had low HDL cholesterol levels. Impaired fasting glucose was found in 10 of 38 patients in which it was measured, and 9 of 25 patients had fasting hyperinsulinemia. Eleven of 37 patients (29.7%) met the diagnosis of the metabolic syndrome. Diastolic BP correlated positively with BMI (r=0.440, *P*=.001), and HDL cholesterol correlated negatively with weight and BMI (r=−0.487, *P*=.002 and r=−0.317, *P*=.05). HOMA-IR correlated positively with BMI and triglyceride levels and negatively with HDL cholesterol levels.

**CONCLUSION:**

Obese Saudi children and adolescents have multiple risk factors associated with metabolic syndrome.

In parallel with the worldwide growing problem of obesity, there have been several reports of an increasing prevalence of childhood obesity in the Saudi population.^1^,^2^ Obesity in childhood is associated with the development of the metabolic syndrome.^3^,^4^ Although the endpoints for cardiovascular risk are not usually seen in childhood, the components of the insulin resistance syndrome (obesity, hypertension, dyslipidemia, and hyperinsulinemia) track from childhood into adulthood.^5^ Several studies have reported a high prevalence of the metabolic syndrome risk factors in obese children and adolescents.^6^,^7^ The prevalence of the metabolic syndrome in overweight adolescents from the United States Third National Health and Nutrition Examination Survey (NHANES III) was 28%.^6^ In addition, 30% of Hispanic overweight youth with family history of type 2 diabetes had the metabolic syndrome.^7^ To date, there are no published studies on the prevalence of the metabolic syndrome risk factors among Saudi children and adolescents. This study is the first to examine the extent of the metabolic syndrome in a group of obese Saudi children and adolescents.

## SUBJECTS AND METHODS

Fifty-seven Saudi children and adolescents with a mean (standard deviation) age of 9.8 (3.5) years were seen in the Pediatric Endocrinology Clinic at King Faisal Specialist Hospital and Research Centre-Jeddah between 2004 and 2008 for evaluation of obesity. We systematically reviewed their medical records and extracted information on age, gender, weight, height, body mass index (BMI), blood pressure (BP), fasting glucose, fasting insulin, and fasting lipid profile. Given the retrospective nature of the study, data were missing for 20 patients for one or more of the biochemical parameters. Obesity was defined as a BMI above the 95^th^ percentile for age and gender.^8^ Exclusion criteria included obesity associated with genetic syndromes, the presence of diabetes, diseases and/or medications that alter BP or lipid metabolism. Hypertension was defined as a systolic BP and/or diastolic BP>95^th^ percentile by gender, age, and height.^9^ Abnormalities in fasting levels of triglycerides, cholesterol, low-density lipoprotein (LDL) cholesterol, and high-density lipoprotein (HDL) cholesterol were adjusted for age and sex (>95^th^ percentile for triglycerides, cholesterol, LDL; and <5^th^ percentile for HDL).^10^ The reference value for elevated fasting plasma glucose (FPG) was taken from the American Diabetes Association guideline of >5.6 mmol/L.^11^ The diagnosis of the metabolic syndrome was defined according to the Third Report of the National Cholesterol Education Expert Panel-Adult treatment Panel (NCEP-ATP III) criteria^12^ and the World Health Organization criteria,^13^ modified for children. The subjects were classified as having the metabolic syndrome if they met three or more of the following criteria for age and sex: BMI>95^th^ percentile, triglyceride level >95^th^ percentile, HDL cholesterol level <5^th^ percentile, systolic or diastolic BP>95^th^ percentile, and impaired fasting glucose. The degree of insulin resistance was estimated (fasting glucose [mmol/L]×fasting insulin [μU/mL]÷22.5) using the homeostasis assessment model- insulin resistance (HOMA-IR) score with higher scores indicating greater insulin resistance.^14^

Data is presented as means (standard deviation). Pearson's correlation analysis was used to study the relationship between variables. Statistical significance was considered at *P*<.05.

## RESULTS

The mean age of the subjects was 9.8 (3.5) years; 33 (57.9%) were males. Mean weight and BMI were 63.7 (28.3) kg and 31.6 (8.0) kg/m^2^, respectively. All subjects had a BMI above the 95^th^ percentile for age and sex. The BMI Z score averaged 3.5 (0.7). Table 1 shows the anthropometric and metabolic characteristics of the subjects. Twenty-four of the 57 children (44%) had a systolic BP above the 95^th^ percentile for age and height, and only two children had a diastolic BP above the 95^th^ percentile for age and height. Of the 39 children who had a lipid profile in their records, 10 (25.6%) had hypertriglyceridemia, 8 (20.5%) had hypercholesterolemia 6 (15.4%) had elevated LDL cholesterol levels, and 6 (15.4%) had low HDL cholesterol levels for age and sex. Impaired fasting glucose was found in 10 of 38 patients (26.3%), and 9 of 25 patients (36%) tested had hyperinsulinemia. Males and females did not differ significantly in any of the anthropometric or biochemical parameters.

**Table 1:** Anthropometric and metabolic characteristics of subjects (n=57).

Characteristic	Mean (SD)	Number (%) of children with abnormalities
Age (years)	9.8 (3.5)	N/A
Weight (kg)	63.7 (28.3)	57/57 (100%)
BMI (kg/m^2^)	31.6 (8)	57/57 (100%)
BMI z score	3.5 (0.7)	57 /57(100%)
Systolic BP (mm Hg)	118.6 (13.2)	24/57 (44%)
Diastolic BP (mm Hg)	67.3 (7.4)	2/57 (3.5%)
Triglycerides (mmol/L)	1.0 (0.5)	10/39 (25.6%)
Total cholesterol (mmol/L)	4.3 (0.9)	8/39 (20.5%)
LDL cholesterol (mmol/L)	2.8 (0.7)	6/39 (15.4%)
HDL cholesterol (mmol/L)	1.3 (0.4)	6/39 (15.4%)
Fasting glucose (mmol/L)	5.2 (0.5)	10/38 (26.3%)
Fasting insulin (pmol/L)	147.6 (101.8)	9/25 (36%)

N/A=not applicable

Among the 37 subjects who had all data available, 15 (40%) had two components of the metabolic syndrome, 6 (16%) had three components, 4 (11%) had four components, and one had all of the five components. Eleven of the 37 subjects (29.7%) met the diagnosis of metabolic syndrome. In addition, 9 of the 20 subjects (45%) with missing biochemical data had two components of the metabolic syndrome. Weight and BMI correlated positively with age (r=0.756, *P*<.001 and r=0.528, *P*<.001 respectively). Also, diastolic BP correlated positively with BMI (r=0.440, *P*=.001). HOMA-IR correlated positively with weight (r=0.513, *P*=.01), BMI (r=0.619, *P*=.001), and triglyceride levels (r=0.461, *P*=.03); and correlated negatively with HDL cholesterol levels (r=−0.481, *P*=.03). HDL cholesterol correlated negatively with weight and BMI (r=−0.487, *P*=.002 and r=−0.317, *P*=.05).

## DISCUSSION

The metabolic syndrome is defined as a cluster of cardiovascular disease risk factors that include abdominal obesity, diabetes and raised fasting glucose concentrations, dyslipidemia, and hypertension.^12^,^13^ Although a uniform definition of the syndrome in pediatrics is lacking, several studies have shown that the syndrome develops in childhood and is prevalent among overweight children and adolescents.^6^,^7^

The prevalence of obesity in Saudi children has been increasing over the past few decades. A recent study of trends in the nutritional status of Saudi children comparing growth data collected between 1993 and 1994 with those collected between 2004 and 2005 from all regions of Saudi Arabia reported a tendency toward overweight and obesity over the last decade.^15^ Another study reported an increase in the proportion of obese schoolboys from 3.4% in 1988 to 24.5% in 2005.^16^

Our study is the first to demonstrate a high prevalence of the metabolic syndrome and its components in a group of obese Saudi children. Thirty percent of obese Saudi children in our study had three or more of the components of the metabolic syndrome and therefore met the criteria for the diagnosis of the metabolic syndrome. The overall prevalence of the metabolic syndrome in adolescents in the United States was 4.2% while the prevalence in obese adolescents was 28.7%.^6^ In addition, 89% of overweight US adolescents had at least one abnormality of the metabolic syndrome and 56% had two components of the metabolic syndrome.^6^ Our data correlate with international data^6^,^7^ and suggest that a substantial number of Saudi children may have the metabolic syndrome. Insulin resistance correlated with the degree of obesity; this emphasizes the central role that obesity plays in the development of insulin resistance. Furthermore, the correlation of insulin resistance with the various components of the metabolic syndrome in our study highlights the importance of insulin resistance in the pathophysiology of the syndrome in childhood.

Hyperinsulinemia, dyslipidemia, hypertension, and impaired fasting glucose seem to be highly prevalent in obese Saudi children. Epidemiological evidence suggests that the clustering of the metabolic syndrome components extends strongly into adult life.^17^ Also, the pathological processes associated with the development of atherosclerotic cardiovascular disease, fatty streaks and fibrous plaques in the aorta and coronary arteries, have been shown to begin during childhood.^18^ With these cardiovascular risk factors manifesting in childhood, earlier presentation of cardiovascular events will emerge as a major and challenging public health problem. Pediatricians need to be alerted regarding the existence of the metabolic syndrome in obese children. In addition, screening for the various components of the syndrome in obese children and early intervention are recommended. Further research is needed to determine the effectiveness of prevention and of various lifestyle and pharmacologic interventions in the management of the metabolic syndrome in children.

Our results have to be interpreted in the context of the limitations of a hospital-based retrospective analysis with some missing data in addition to a small sample size. A large population based study is recommended to establish the prevalence of the metabolic syndrome and its components in Saudi obese and nonobese children.
